# Reprocessing of Medical Products in Electrophysiology

**DOI:** 10.5935/abc.20170010

**Published:** 2017-02

**Authors:** Ricardo Ryoshim Kuniyoshi, Eduardo Back Sternick, Elenir Nadalin, Denise Tessariol Hachul

**Affiliations:** Sociedade Brasileira de Arritmias Cardíacas, São Paulo, SP - Brazil

**Keywords:** Recycling, Electrophysiologic Techniques / cardiac, Catheter Ablation, Cost Savings

Electrophysiological procedures use high-cost multipolar electrode catheters which can be reprocessed. The reuse thereof has been performed by electrophysiology services in Europe, United States, Latin America and also in our midst. In fact, prior studies have proved that there is an actual cost decrease^1,2^ and have also attested to the safety and efficacy of such practice,^3-12^ observing rates of complication and therapeutic results similar to the ones obtained with first-use electrophysiology devices. The growing concern with sustainability and no waste, associated with the efficacy and safety already demonstrated, increasingly stimulate the practice of reprocessing single-use medical devices throughout the world.

The American Society of Cardiac Arrhythmias issued a favorable opinion to the reprocessing of electrophysiological devices to the FDA - Food And Drug Adminstration^13^ as did the GAO - Government Accountability Office, a federal oversight entity of the United States.^14^

In Brazil, the reprocessing of such products was regulated by the National Health Surveillance Agency (ANVISA), through a Resolution by the Collegiate Board (RDC) 156^15^ and Special Resolution (RE) 2605,^16^ both published in 2006. The RDC 156 establishes that the authorization of reprocessing single-use medical devices, should be at the time of registration in Brazil.^15^ Despite the fact that most of the manufacturers labeled their products as single-use, ANVISA demands the submission of documents that substantiate the reasons for not reprocessing. Once the manufacturer's arguments are proved and accepted, the words "Reprocessing Forbidden" must be included in the label of that certain product. Also, RE 2605^16^ lists 66 materials classified as materials whose reprocessing is invariably forbidden. We stress that said list does not contain any product used in the electrophysiological procedures routine.

In 2013, ANVISA issued Technical Note No. 001/2013^17^ reiterating the validity of the reprocessing rules published in 2006, in reply to the users' recurrent doubts and demands for clarifications, as per the following excerpt from the resolution: "*demands and questions regarding the correct interpretation to be given to the contents of the labels of product for a single use, available in the market, has become increasingly frequent*". Currently, in spite of this notice, doubts still persist with regard to the understanding of the rules in force. Due to that, we have made a detailed analysis of the labels of materials routinely used here in electrophysiology procedures, with the purpose of assessing possible incongruences that justify misunderstandings and interpretation errors.

For such analysis, we analyzed the contents of the labels of materials used in electrophysiological procedures, written in Portuguese, available in ANVISA's database http://www.anvisa.gov.br/scriptsweb/correlato/correlato_rotulagem.htm. We included labels from 7 manufacturers that registered products intended for electrophysiology with ANVISA. Once the website had been accessed, we typed the name of the manufacturers in field "*Supplier's Name*", obtaining a complete list of medical products each manufacturer. Afterwards, we chose only the labels of the products used in electrophysiological procedures. The labels were then printed out, numbered and grouped according to their similarity with regard to physical characteristics and technical applicability, classified as: 1) fixed-curve diagnostic catheter; 2) deflectable-curve diagnostic catheter; 3) circular catheter or high-density mapping catheter; 4) non-irrigated ablation catheter; 5) irrigated ablation catheter; 6) introducers and sheaths; 7) transseptal needle; and 8) intracardiac echocardiography catheter. Labels and/or records with more than one kind of product were attached more than once, that is, one for each of the products to which they corresponded, according to the applicability and characteristic thereof.

The products were then classified into 5 groups:

1. G1 - reprocessing permitted;
2. G2 - reprocessing forbidden;
3. G3 - irregular condition, for not complying with the labeling recommendations set forth in RDC 156;
4. G4 - conflicting information; and
5. G5 - no label in ANVISA's database.

This classification was based on the RDC 156, which recommends that the labels should contain only the words: "Reprocessing Forbidden" or "The manufacturer recommends single use"^15^. Thus, the products whose labels did not contain the expression "Reprocessing Forbidden" were defined as G1, and they may or may not contain the words "The manufacturer recommends single use". In G2, the products whose labels carried the expression "Reprocessing Forbidden" were included, in spite of the presence of any other word or information. In G3, labels with expressions "Single Use", "Product for Single Use", "Do Not Re-Sterilize", "Discard after using" and "Destroy after using" were included, even if accompanied by expression "The manufacturer recommends single use", given that, pursuant to Technical Note No. 001/2013,^17^ these sentences are considered to not be in conformity with the rules of the regulatory agency. The products that had 2 or more labels, with recommendations differing from one another and/or irregular, were classified as G4. And lastly, the products not in ANVISA's database were classified as G5.

The products included in G1 and G2 were considered to have their labels in conformity with ANVISA's rules, while those classified as G3, G4 and G5 were considered to not be in conformity.

For each group of products with the same applicability and characteristic, and whose labels were in conformity with ANVISA (G1 and G2), it was also assessed whether they were uniform with regard to reprocessing prohibition or not.

Lastly, physical labels were compared by sampling with the labels in ANVISA's database, to assess whether the information contained in both sources matched.

The labeling research was made from July 25, to August 25, 2016 and identified 121 products used in electrophysiological procedures, registered with ANVISA's database, totaling 116 labels (Table 1). Forty-five labels (37.2%) were classified as reprocessing permitted (G1); 41 (33.9%) as reprocessing forbidden (G2); 28 (23.1%) were irregular (G3); 3 (2.5%) had two or more labels with conflicting information (G4) and lastly, four products (3.3%) did not have labels in ANVISA's database (G5). We were then able to note that 86 (71.1%) labels were in conformity, whereas 34 (28.9%) were not in conformity with RDC 156.

**Table 1:** Labels of medical products grouped according to similarity of characteristics and applicability

Medical products in electrophysiology	In conformity with RDC 156		Not in conformity with RDC 156			Total
	G1	G2	G3	G4	G5	
	1 (20%)	4 (80%)	0	0	0	5
Non-irrigated Ablation Catheter	7 (36.8%)	4 (21%)	7 (36.8%)	1 (5.3%)	0	19
Irrigated ablation catheter	9 (33.3%)	15 (55.5%)	3 (11.1%)	0	0	27
Deflectable-curve diagnostic catheter	9 (50%)	4 (22.2%)	3 (16.7%)	1 (5.5%)	1 (5.5%)	18
Fixed-curve diagnostic catheter	2 (22.2%)	4 (44.4%)	1 (11.1%)	1 (11.1%)	1 (11.1%)	9
High-density mapping circular catheter	7 (58.3%)	2 (16.7%)	2 (16.7%)	0	1 (8.3%)	12
Introducers and sheaths	4 (16%)	8 (32%)	12 (48%)	0	1 (4%)	25
Intracardiac echocardiography catheter	6 (100%)	0	0	0	0	6
Total	45 (37.1%)	41 (33.9%)	28 (23.1%)	3 (2.5%)	4 (3.3%)	121 (100%)

G1: reprocessing permitted; G2: reprocessing forbidden; G3: irregular in relation to the recommendations in RDC 156; G4: conflicting information; G5: labels absent from ANVISA’s database.

The analysis of sub-groups of products with similar characteristics and the same applicability, included in G1 and G2 (labels in conformity with RDC 156), showed that only the intracardiac echocardiography catheter was uniform with regard to the reprocessing recommendations. In this specific case, all six existing types had in their labels the words "The manufacturer recommends single use", which characterizes, therefore, a reprocessing permission. Other products did not have parity in the contents of their labels (Table 1).

Three products were classified as G4, of which one was a fixed-curve diagnostic catheter, one was a deflectable-curve diagnostic catheter and another a non-irrigated ablation catheter with bidirectional curve, all of which were from different manufacturers. The three products had more than one label catalogued in ANVISA's database, under the same registration number and with different recommendations. For the fixed-curve diagnostic catheter (ANVISA registration 10192030102), three labels were found, with the following information: "Reprocessing Forbidden", "The manufacturer recommends single use" and "Product for Single Use", which, pursuant to the RDC 156, mean, respectively, reprocessing forbidden, reprocessing allowed and irregular information. The deflectable-curve diagnostic catheter (ANVISA registration 10341350368) and the ablation catheter (ANIVISA registration 10332340206), for their turn, had two labels, with the following words: "Reprocessing Forbidden" and "The manufacturer recommends single use", which are contradictory instructions.

Lastly, nine physical labels of products used in electrophysiology (Table 2) were analyzed. In six of them, no reprocessing information was found. Upon assessing these six labels in ANVISA's database, we were able to ascertain that one of them contained the expression "The manufacturer recommends single use"; three of them contained the words "Reprocessing Forbidden", and in the other one, the information was not in conformity with RDC 156. In addition, one product (transseptal introducer sheath, registered with ANVISA under No. 10332340208) did not have a label in ANVISA's database.

**Table 2:** Comparative analysis of physical labels vs. labels in ANVISA’s database

Product	ANVISA Registration	Physical Label	Label in the Website
Non-irrigated Ablation Catheter	10332340098	The manufacturer recommends single-use	The manufacturer recommends single-use
Deflectable Diagnostic Catheter	10332340161	The manufacturer recommends single use	The manufacturer recommends single use
Circular Catheter	10332340332	No expression	Product for a single use
Irrigated Ablation Catheter	10332340361	No expression	The manufacturer recommends single use
Deflectable introducer	10332340207	No expression	Product for a single use Reprocessing forbidden Destroy after using
Non-irrigated Ablation Catheter	10332340226	The manufacturer recommends single use	The manufacturer recommends single use
Transseptal Needle	10332340151	No expression	Product for a single use Reprocessing forbidden Destroy after using
Hemostatic Introducer	10332340107	No expression	Product for a single use Reprocessing forbidden Destroy after using
Transseptal Introducer	10332340208	No expression	Not found

As we were able to note in this analysis, in spite of the fact that the reprocessing of materials used in electrophysiological procedures is allowed and regulated by ANVISA, there are important incongruences in the labels, in a number of products that is not trifling, which may generate mistaken interpretations by the users, and consequently the improper reprocessing of said materials.

The contents of 34 labels (28.9%) from ANVISA's database, which are not in conformity with RDC 156, require urgent adaptation.

We consider it to be extremely important for this information, defined upon the registration of the product, to be clear and irrefutable, thus making sure a quick and correct indentification of medical products with regard to the use thereof. In this regard, the labels should contain a single expression, clearly defining the situation of each medical product: "reprocessing forbidden" or "reprocessing allowed". We are also of the opinion that the criteria used to classify the product must be standardized and ensure the equity of information contained in the physical labels and in ANVISA's database.

Also, it is our opinion that the technical information submitted to ANVISA by the manufacturers, in justification of the prohibition of reprocessing a certain product, upon the registration thereof, must be accessible to the users for them to be aware of it.

For these reasons, the Brazilian Society of Cardiac Arrhythmias (SOBRAC) met with the suppliers of electrophysiological products available in the domestic market and suggested an immediate review of the information contained in the labels, so as to adapt them to ANVISA's standards and make the information unequivocal.

In summary, after the conduction of this research, it was possible to reach the following conclusions and suggestions:

1. The reprocessing and reuse of medical products in electrophysiology is permitted in Brazil and regulated by ANVISA, through RDC 156;
2. A thorough analysis of the labels found inconsistencies that could entail misinterpretations and improper decisions by the users with regard to the compliance with RDC 156, even if unintentional.
3. In the current scenario, while these incongruences are not rectified, it is necessary for the healthcare services that reprocess such products to make a stringent and systematic assessment of both product labels: the physical one and the one in ANVISA's database, with the purpose of identifying the ones that are not in conformity with the RDC 156, as well as those that contain differing instructions, thus avoiding mistakes in the compliance with ANVISA's orders.
